# Artificial Intelligence in Transcranial Doppler Ultrasonography

**DOI:** 10.2174/0115734056331493241217075436

**Published:** 2025-01-17

**Authors:** Antonio Siniscalchi, Vincenzo Inghingolo, Piergiorgio Lochner, Giovanni Malferrari

**Affiliations:** 1 Department of Neurology and Stroke Unit, Azienda Ospedaliera di Cosenza, Cosenza, Italy; 2 Neurology Unit and Stroke Unit, Department of Medicine, Casa Sollievo della Sofferenza, San Giovanni Rotondo, Italy; 3 Department of Neurology and Stroke Unit, Saarland University, Medical Center, Homburg, Germany; 4 UOC Neurologia e Rete Stroke Metropolitana, Istituto delle Scienze Neurologiche di Bologna, Bologna, Italy

**Keywords:** Antificial intelligence, Transcranial doppler, Transcranial duplex code color, Neurosonology, Cerebral hemodynamics, Ultrasonography

## Abstract

Transcranial Doppler is an instrumental ultrasound method capable of providing data on various brain pathologies, in particular, the study of cerebral hemodynamics in stroke, quickly, economically, and with repeatability of the data themselves. However, literature reviews from clinical studies and clinical trials reported that it is an operator-dependent method, and the data can be influenced by external factors, such as noise, which may require greater standardization of the parameters. Artificial intelligence can be utilized on transcranial Doppler to increase the accuracy and precision of the data collected while decreasing operator dependencies. In a time-dependent pathology, such as stroke, characterized by hemodynamic evolution, the use of artificial intelligence in transcranial Doppler ultrasound could represent beneficial support for better diagnosis and treatment in time-dependent pathologies, such as stroke.

## 1 INTRODUCTION

Transcranial insonation of the cerebral circulation has been indicated in several pathologies, including ischemic stroke due to large vessel occlusion [1], elevated intracranial pressure [2], traumatic brain injury [3], and dementia [4]. Currently, there are two types of transcranial Doppler devices: transcranial Doppler (non-duplex, TCD) and later color-coded transcranial duplex TCD (TCCD) [1, 4]. TCDs (non-duplex, non-imaging devices) are capable of detecting arteries with the use of audible shift and Doppler spectrum without any need for visual guidance [1, 4]. TCCD (duplex imaging equipment) is an ultrasound technique that uses 2-3 MHz transducers and software to provide images of the cerebral vessels and simultaneously the Doppler spectrum of the same [1, 4]. Both TCD/TCCD are rapid, economical, non-invasive, and repeatable ultrasound diagnostic methods capable of providing real-time assessment of the flow of large cerebral arteries [1] through the four most commonly used acoustic windows: temporal, suboccipital, transorbital, and submandibular, although bone thickness, which is a patient-dependent limitation, may prevent acoustic window insonation by 10-15%, especially in blacks, Asians, and older women. This may be related to the thickness and porosity of bone around the acoustic windows and the attenuation of ultrasonic energy transmission [4-7]. Recently, the high sensitivity of the MicroV instrument has been found to allow acoustic window insonation even with suboptimal windows [5].

## 2 TRANSCRANIAL DOPPLER AND CEREBRAL BLOOD FLOW

The use of the Doppler signal of the cerebral arteries allows us to obtain haemodynamic parameters in both physiological and pathological conditions (*e.g*., stenosis, vasospasm, monitoring in emboli, management of cerebral arteriovenous malformations) [5, 6]. Although TCD/TCCD ultrasound provides a measure of the hemodynamic status of the cerebral blood vessels, it cannot provide an exact angle of incidence for soundproofing of the Doppler spectrum. The most accurate measurement of the flow velocity of a vessel is when the sounding angle is zero; that is, the probe is parallel to the direction of flow since the cosine of 0 is 1 [5]. When the angle is larger and the cosine value is smaller, there is a greater chance of a speed measurement error. In order to maintain a 15% error, it is essential to lower this angle to less than 30 degrees [7]. Therefore, it is necessary for doctors to know the anatomy and the course of the intracranial blood vessels and to be able to perform qualified ultrasound examinations, for example, to better define intracranial stenosis. The most commonly used key parameters in TCD/TCCD are detection depth, blood flow velocity peak systolic velocity (PSV), end-diastolic velocity (EDV), mean flow velocity, MFV), pulsability index (PI) and resistance index (RI), and characteristic spectral index of blood flow (the intensity of the doppler spectral signal is represented by the color signal that goes from weak to strong with a progressive color gradation from blue, to yellow and to red) [4, 5]. An important limitation of TCD/TCCD ultrasound is that the operator does not accurately visualize the course of the intracranial vessels and has difficulty establishing the best angle of incidence for vessel sounding, reducing the accuracy of repeated measurements of vessel blood flow velocity [8]. This means that this instrumental examination is operator-dependent.

## 3 ARTIFICIAL INTELLIGENCE AND TRANSCRANIAL DOPPLER

Artificial intelligence is a branch of computer science that aims to create a new intelligent device that is as similar to human intelligence as possible. Artificial intelligence is comprised of robotics, speech recognition, image recognition, natural language processing, expert systems, *etc* [9]. Significant changes in the medical field have been caused by the rapid development of artificial intelligence. Artificial intelligence can improve data collection. Artificial intelligence, using computer science techniques, can make clinical diagnoses and provide treatment recommendations. It has been widely applied in many clinical conditions to diagnose, treat, and predict outcomes [10]. It is increasingly becoming applicable and useful to the whole of healthcare [11] and has been shown to have important use in emergency medicine, particularly for acute radiological imaging [12]. Artificial intelligence can be used for TCD/TCCD ultrasound, as evidenced by recent studies. It can determine a testing system that combines the brain-computer interface (BCI) and TCD/TCCD ultrasound [12]. This can provide dramatic information about changes in cerebral blood flow velocity in relation to mental activities, such as the creation of words and the rotation of the brain. By utilizing artificial intelligence algorithms, we can classify and detect artifacts in TCD/TCCD ultrasound signals using artificial intelligence algorithms [13]. Ultrasound signals, as in other methods used in medicine, are often altered by noise artifacts from sources, such as interference from power lines and interference from the movement of recording electrodes. The authenticity of TCD/TCCD ultrasonic signals requires effective noise removal. A robotic ultrasound system has recently been developed that combines ultrasound, robotics, and machine learning. This system combines TCD/TCCD with a square cuff containing a robotic rod that allows for automatic adjustment of the ultrasound probe for better visualization and recording of hemodynamic parameters of intracranial cerebral vessels. Furthermore, the feasibility and accuracy of robot-assisted TCD to measure cerebral blood flow velocities in the anterior and MCA in patients with subarachnoid hemorrhage and vasospasm were studied [14, 15]. The system also determines a better soundproofing angle of the probe even after the patient has moved to continue sonicating at previously acquired positions [14].

Although TCD/TCDD has benefits for the study of cerebral hemodynamics (in terms of speed of execution, cost-effectiveness in screening and in perioperative monitoring, and repeatability), especially in ischemic stroke in detecting stenosis or occlusion of intracranial vessels [16], we have observed some limitations in its use [17, 18]. These limitations are mainly determined by the fact that ultrasound techniques on cerebral vessels are highly operator-dependent since the ultrasound method requires a three-dimensional knowledge of the anatomy and course of the cerebral vessels [17, 18]. Furthermore, the recorded speed measurements can be influenced by other factors, such as the sounding angle, noise, and differences in the partial pressure of CO2 in the blood. Potential future applications of artificial intelligence and machine learning may guide TCD/TCCD systems towards better visualization and acceptable image quality and measurement conditions that will not only aid in decision-making but also improve clinical ultrasound workflow and reduce healthcare costs.

## CONCLUSION

All these conditions mean that artificial intelligence can help in the data collection process of the instrumental ultrasound method in a more selective way, requiring less dependence on the operator and better accuracy of the data obtained. The use of artificial intelligence in TCD/TCCD can better assist doctors in the diagnosis and treatment of a time-dependent brain pathology, such as a stroke, characterized by its own hemodynamic evolution.

## LIST OF ABBREVIATIONS

BCI: Brain-computer interface
PSV: Peak systolic velocity
EDV: End-diastolic velocity
MFV: Mean flow velocity
PI: Pulsability index
RI: Resistance index
